# Supplementary material for: The effect of metabolic health and obesity on lung function: A cross sectional study of 114,143 participants from Kangbuk Samsung Health Study
Source: PLoS One. 2022 Apr 13;17(4):e0266885. doi: 10.1371/journal.pone.0266885 (PMC9007386; doi:10.1371/journal.pone.0266885)
Supplement: S1 Table — (DOCX) [file pone.0266885.s001.docx]

**S1 Table. Multiple logistic regression analysis of impaired spirometric parameters according to metabolic health defined as absence of any component of metabolic syndrome and obesity status.**

|  | **Model 1** | | | **Model 2** | | | **Model 3** | | |
| --- | --- | --- | --- | --- | --- | --- | --- | --- | --- |
|  | **OR (95% CI)** | ***p* value** | ***p* for trend** | **OR (95% CI)** | ***p* value** | ***p* for trend** | **OR (95% CI)** | ***p* value** | ***p* for trend** |
| FEV1%<80% |  |  | <0.001 |  |  | <0.001 |  |  | 0.001 |
| MHNO (reference) | 1 |  |  | 1 |  |  | 1 |  |  |
| MHO | 0.816 (0.681-0.977) | 0.027 |  | 0.787 (0.653-0.948) | 0.012 |  | 0.785 (0.621-0.946) | 0.011 |  |
| MUHNO | 1.268 (1.149-1.399) | <0.001 |  | 1.198 (1.081-1.328) | 0.001 |  | 1.191 (1.073-1.323) | 0.001 |  |
| MUHO | 1.257 (1.105-1.431) | 0.001 |  | 1.115 (0.972-1.279) | 0.118 |  | 1.106 (0.961-1.272) | 0.159 |  |
| Metabolic unhealthy | 1.323 (1.215-1.441) |  | <0.001 | 1.236 (1.130-1.352) |  | <0.001 | 1.231 (1.123-1.350) |  | <0.001 |
| Obesity | 0.847 (0.793-0.903) |  | <0.001 | 0.904 (0.828-0.986) |  | 0.022 | 0.902 (0.820-0.993) |  | 0.035 |
| FVC%<80% |  |  | <0.001 |  |  | <0.001 |  |  | <0.001 |
| MHNO (reference) | 1 |  |  | 1 |  |  | 1 |  |  |
| MHO | 0.579 (0.463-0.723) | <0.001 |  | 0.545 (0.431-0.687) | <0.001 |  | 0.542 (0.429-0.684) | <0.001 |  |
| MUHNO | 1.255 (1.090-1.444) | 0.002 |  | 1.151 (0.992-1.337) | 0.064 |  | 1.136 (0.975-1.322) | 0.102 |  |
| MUHO | 1.197 (1.076-1.331) | 0.001 |  | 1.147 (1.026-1.282) | 0.016 |  | 1.137 (1.015-1.273) | 0.026 |  |
| Metabolic unhealthy | 1.347 (1.228-1.477) |  | <0.001 | 1.289 (1.168-1.421) |  | <0.001 | 1.279 (1.158-1.414) |  | <0.001 |
| Obesity | 0.863 (0.804-0.926) |  | <0.001 | 0.862 (0.785-0.947) |  | 0.002 | 0.835 (0.752-0.928) |  | 0.001 |
| FEV1(L)/FVC(L) ratio<0.7 |  |  | 0.182 |  |  | 0.601 |  |  | 0.895 |
| MHNO (reference) | 1 |  |  | 1 |  |  | 1 |  |  |
| MHO | 1.026 (0.851-1.237) | 0.789 |  | 1.106 (0.908-1.347) | 0.315 |  | 1.144 (0.935-1.399) | 0.191 |  |
| MUHNO | 0.905 (0.710-1.153) | 0.419 |  | 0.938 (0.730-1.205) | 0.615 |  | 0.958 (0.750-1.233) | 0.741 |  |
| MUHO | 1.244 (1.048-1.478) | <0.013 |  | 1.285 (1.074-1.537) | 0.006 |  | 1.298 (1.085-1.554) | 0.004 |  |
| Metabolic unhealthy | 1.127 (0.993-1.279) |  | 0.065 | 1.116 (0.981-1.271) |  | 0.096 | 1.094 (0.958-1.250) |  | 0.183 |
| Obesity | 1.371 (1.212-1.550) |  | <0.001 | 1.139(1.181-1.517) |  | <0.001 | 1.295(1.131-1.484) |  | <0.001 |

Model 1 was adjusted for age, sex, smoking status, alcohol intake, regular exercise and education level. Model 2 was adjusted as in model 1 plus systolic BP, glucose, total cholesterol, triglycerides, high-density lipoprotein cholesterol, low density lipoprotein cholesterol, and HOMA-IR. Model 3 was adjusted as in model 2 plus variables with a *p* <0.05 in univariate analyses.

CI=confidence interval; FEV1%=percent predicted forced expiratory volume in 1s; FVC%=percent predicted forced vital capacity; MHNO=metabolically healthy non-obese; MHO=metabolically healthy obese; MUHNO=metabolically unhealthy non-obese; MUHO=metabolically unhealthy obese; OR=odds ratio.
