# Supplementary material for: The effect of metabolic health and obesity on lung function: A cross sectional study of 114,143 participants from Kangbuk Samsung Health Study
Source: PLoS One. 2022 Apr 13;17(4):e0266885. doi: 10.1371/journal.pone.0266885 (PMC9007386; doi:10.1371/journal.pone.0266885)
Supplement: Attachment — Submitted filename: Response to Reviewers.docx [file pone.0266885.s002.docx]

Manuscript ID: PONE-D-21-23871

**Title: Metabolically-healthy obesity is not detrimental to lung function: A Kangbuk Samsung Health Study**

The authors appreciate the time and effort given by all of the reviewers and the editor in reviewing this manuscript. The constructive criticism has been well-received by all of us. Thank you for the constructive comments and criticisms of our manuscript named above. In response to the reviewer’s suggestions and questions, we have revised the manuscript as suggested. We have responded to the reviewer’s comments in a point-by-point manner below. Please find enclosed the revised version of the manuscript. We hope we have dealt with comments of the reviewers to their satisfaction. We hope that this work is now suitable for publication and look forward to your response.

Your assistance will be greatly appreciated.

With best wishes

Jae-Uk Song, MD, PhD,

Division of Pulmonary and Critical Care Medicine, Department of Internal Medicine, Kangbuk Samsung Hospital, Sungkyunkwan University School of Medicine,

29 Saemunan-ro, Jongno-gu, Seoul 03181, Republic of Korea

Phone: +82-2-2001-1597

Fax: +82-2-2001-1596

E-mail: [khfever76@gmail.com](mailto:khfever76@gmail.com)

**Point-by-point responses to reviewer’s comments**

**TO THE COMMENTS OF ADDITIONAL EDITOR.**

**C1)**

When submitting your revision, we need you to address these additional requirements.

1. Please ensure that your manuscript meets PLOS ONE's style requirements, including those for file naming. The PLOS ONE style templates can be found at

<https://journals.plos.org/plosone/s/file?id=wjVg/PLOSOne_formatting_sample_main_body.pdf> and

<https://journals.plos.org/plosone/s/file?id=ba62/PLOSOne_formatting_sample_title_authors_affiliations.pdf>.

**R1)**

Thank you for pointing out the lack of clarity. We apologize for our mistakes and carelessness. We revised our manuscript which meets PLOS ONE's style requirements. We reflected additional editor’s recommendations, as seen from the modified manuscript file attached to this “Response to Reviewers”. Once again, we apologize for our carelessness and we really appreciate your concern.

We sorted title centrally and capitalize only the first word of the title except for proper noun. (Kangbuk Samsung Health Study) But, we also modified title as suggestion by reviewer 1.

**(from line 1 to line 2 on page 1) in the revised manuscript**

**Metabolically-healthy obesity is not detrimental to lung function: A Kangbuk Samsung Health Study**

**Short title:** Metabolically-healthy obesity and lung function

🡪

**The effects of metabolically health and obesity on lung function:**

**a cross sectional study of 114,143 participants from Kangbuk Samsung Health Study**

**Short title:** Metabolically-healthy obesity and lung function

We also modified “Key words” subheading section in abstract

**(from line 48 to line 49 on page 3) in the revised manuscript**

**Key Words:** Lung function, Obesity, Metabolic syndrome, spirometry, metabolically heathy obesity

🡪

**Key words:** lung function, obesity, metabolic syndrome, spirometry, metabolically heathy obesity

**(line 158 on page 8) in the revised manuscript**

We fixed subheading, “**Statistical Analyses**” into “**Statistical analyses**” in method section.

**(from line 185 on page 9) in the revised manuscript**

We changed heading, “**RESULTS**” into “**Results**”.

We also used a larger font of subheading (from 12pt to 16p) for consistency of context.

We modified subheading in result section as follow:

**(from line 238 on page 19) in the revised manuscript**

**Odd ratios (ORs) for Impairment of Lung Function**

🡪

**Odd ratios (ORs) for impairment of lung function**

We additionally modified conclusions in discussion section, as follow:

Some sentences was modified as reviewer#1’s suggestion

**(from line 373 to line 379 on page 27) in the revised manuscript**

In conclusion, we found that MHO was associated with attenuated FVC and FEV1 decline in a middle-aged Korean sample. The association seems to be related to the obesity paradox, the effect of which is particularly strong in our cohort. These findings indicate that MHO is not always harmful, depending on the demographics of the study population. However, longitudinal follow-up studies and prospective interventional studies are required to validate any cause-and-effect relationships between MHO and lung function.

🡪

**Conclusions**

We found that MHO was associated with attenuated FVC and FEV1 decline in a middle-aged Korean sample. The association seems to be related to the obesity paradox, the effect of which is particularly strong in our cohort. These findings suggest maintaining of healthy weight and life style could be more important to improve lung function, than obesity defined by only BMI. However, longitudinal follow-up studies and prospective interventional studies should be required to validate any cause-and-effect relationships between MHO and lung function

Lastly, we saved figure files as “Fig 1.tif “, instead of “Figure 1.tif “.

**C2)**

Thank you for stating the following in the Acknowledgments Section of your manuscript:

“This work was supported by 2022 scientific promotion program funded by Jeju National University”

We note that you have provided funding information that is not currently declared in your Funding Statement. However, funding information should not appear in the Acknowledgments section or other areas of your manuscript. We will only publish funding information present in the Funding Statement section of the online submission form.

Please remove any funding-related text from the manuscript and let us know how you would like to update your Funding Statement. Currently, your Funding Statement reads as follows:

“One of authors (Jonghoo Lee) was supported by 2022 scientific promotion program funded by Jeju National University”

Please include your amended statements within your cover letter; we will change the online submission form on your behalf.

**R2)**

We apologize for our carelessness. Thank you for pointing our mistakes.

We removed funding related sentences in Acknowledgement section. And, also we would be grateful for your kind technical help on updating our funding statement as follows;

One of authors (Jonghoo Lee) was supported by 2022 scientific promotion program funded by Jeju National University. However, the funders had no role in study design, data collection and analysis, decision to publish, or preparation of the manuscript.

**C3)**

We note that the grant information you provided in the ‘Funding Information’ and ‘Financial Disclosure’ sections do not match.

When you resubmit, please ensure that you provide the correct grant numbers for the awards you received for your study in the ‘Funding Information’ section.

R3)

We apologize for difference between ‘Funding Information’ and ‘Financial Disclosure’. We provide the correct grant numbers for the funding that Jonghoo Lee received in the ‘Funding Information’ section. Thank you for pointing our mistakes

**C4)**

In your Data Availability statement, you have not specified where the minimal data set underlying the results described in your manuscript can be found. PLOS defines a study's minimal data set as the underlying data used to reach the conclusions drawn in the manuscript and any additional data required to replicate the reported study findings in their entirety. All PLOS journals require that the minimal data set be made fully available. For more information about our data policy, please see <http://journals.plos.org/plosone/s/data-availability>.

Upon re-submitting your revised manuscript, please upload your study’s minimal underlying data set as either Supporting Information files or to a stable, public repository and include the relevant URLs, DOIs, or accession numbers within your revised cover letter. For a list of acceptable repositories, please see <http://journals.plos.org/plosone/s/data-availability#loc-recommended-repositories>. Any potentially identifying patient information must be fully anonymized.

Important: If there are ethical or legal restrictions to sharing your data publicly, please explain these restrictions in detail. Please see our guidelines for more information on what we consider unacceptable restrictions to publicly sharing data: <http://journals.plos.org/plosone/s/data-availability#loc-unacceptable-data-access-restrictions>. Note that it is not acceptable for the authors to be the sole named individuals responsible for ensuring data access.

We will update your Data Availability statement to reflect the information you provide in your cover letter.

**R4)**

We really appreciate for your consideration. All relevant data are within the paper. However, the data are not available to be shared publicly due to ethical restrictions imposed by the Institutional Review Board of Kangbuk Samsung Hospital, because we do not have a permission from the IRB to distribute the data. The datasets generated and/or analysed during the current study are available through the corresponding author only on permission from the IRB. Only after permission from IRB, we can approach heath screening data inside computer network (called “Cohort VDI system”) only in our hospital. And also, Cohort VDI system has been blocked to download raw data outside network. So, it is impossible to leak our data to the outsiders. This is way beyond my jurisdiction.

Also, we answered to same requests in previous published our works in your journal.

[PLoS One.](https://www.ncbi.nlm.nih.gov/pubmed/30673698) 2019 Jan 23; 14(1):e0208736. “Decreased lung function is associated with risk of developing non-alcoholic fatty liver disease: A longitudinal cohort study.”

PLoS One 2020 Apr 2; 15(4):e023105. “Decreased lung function is associated with elevated ferritin but not iron or transferrin saturation in 42,927 healthy Korean men: A cross-sectional study.”

We hope you understand our policy imposed on our data.

Therefore, we would like you to update our Data Availability statement as follows;

Due to ethical restrictions imposed by the Institutional Review Board of Kangbuk Samsung Hospital, the patient

data are not available for distribution outside of the Kangbuk Samsung Hospital. For additional information, please contact Gayoung Lim (gayoung.lim@samsung.com) or the Institutional Review Board of Kangbuk Samsung Hospital (contact information below).

Address: 29 Saemunan-ro, Jongno-Gu, Seoul, Korea (03181)

E-mail: irb. kbsmc@samsung.com

Telephone: 82-2-2001-1943, 1945 - Fax: 82-2-2001-1946

For your information, we attached some capture images related to Cohort VDI system, just below this sentence;

**TO THE COMMENTS OF THE REVIEWER 1.**

Song and colleagues address whether metabolically healthy obesity influences lung functions based on a cohort of 114,143 people recruited by a cross-sectional study from a medical health checkup program in South Korea. The participants were divided into four groups: MHNO (as reference), MHO, MUHNO and MUHO. Results suggested that MHO group had better lung functions compared with other groups. The study was generally well conducted. Comments are provided as follow:

**Major comment**

**C1)**

The title and conclusion are not supported by the study design. The authors suggested that MHO group is not always harmful, but this is solely based on cross-sectional data. It is likely reverse causation may play a role here. The discussion suggested the use of asymptomatic young individuals minimized the possibility of reverse causation which is not completely convincing. E.g., people with subclinical lung disease may also lose weight because of those diseases. Therefore, I strongly recommend the title and conclusion to be revised to be more neutral.

**R1)**

We do appreciate reviewer’s advice and suggestions with the greatest care. We agree with reviewer’s opinion. As reviewer recommended, we changed the title as follows;

**(from line 1 to line 2 on page 1) in the revised manuscript**

Metabolically-healthy obesity is not detrimental to lung function: A Kangbuk Samsung Health Study

🡪

The effects of metabolically health and obesity on lung function: A cross sectional study of 114,143 participants from Kangbuk Samsung Health Study

We also removed sentence leading to confusion at conclusion section in abstract;

**(from line 46 to line 47 on page 2) in the revised manuscript**

We found that the MHO group had better lung function than other groups. This suggests that MHO is not always harmful. However, longitudinal follow-up studies will be required to validate our findings.

🡪

We found that the MHO group had better lung function than other groups. However, longitudinal follow-up studies will be required to validate our findings.

We additionally modified conclusions in the revised manuscripts, too.

**(from line 373 to line 379 on page 27) in the revised manuscript**

In conclusion, we found that MHO was associated with attenuated FVC and FEV1 decline in a middle-aged Korean sample. The association seems to be related to the obesity paradox, the effect of which is particularly strong in our cohort. These findings indicate that MHO is not always harmful, depending on the demographics of the study population. However, longitudinal follow-up studies and prospective interventional studies are required to validate any cause-and-effect relationships between MHO and lung function.

🡪

**Conclusions**

We found that MHO was associated with attenuated FVC and FEV1 decline in a middle-aged Korean sample. The association seems to be related to the obesity paradox, the effect of which is particularly strong in our cohort. These findings suggest maintaining of healthy weight and life style could be more important to improve lung function, than obesity defined by only BMI. However, longitudinal follow-up studies and prospective interventional studies should be required to validate any cause-and-effect relationships between MHO and lung function.

**Minor comments**

**C1)**

The OR values should be included in the Abstract.

**R1)**

Thank you for the reviewer’s comment. As reviewer recommended, we described aOR values in abstract section as follows;

**(from line 40 to line 44 on page 2) in the revised manuscript.**

Compared with MHNO, the aORs for FVC% < 80% was 0.704 in the MHO group, 1.241 in the MUHNO group, and 1.226 in the MUHO group (p for trend = 0.013). Similar results for FEV1% <80% were also observed (P for trend = 0.014). However, the aORs for FEV1/FVC < 0.7 was not significantly different between groups (p for trend = 0.173).

🡪

Compared with MHNO, the aORs (95% confidence interval) for FEV1% < 80% in MHO, MUHNO and MUHO were 0.871 (0.775-0.978), 1.274 (1.114-1.456), and 1.176 (1.102-1.366), respectively (P for trend = 0.014). Similarly, the aORs in MHO, MUHNO and MUHO were 0.704 (0.615-0.805), 1.241 (1.075-1.432), and 1.226 (1.043-1.441) for FVC% < 80% (p for trend = 0.013).

**C2)**

Sensitivity analysis of stricter definition criteria of metabolic health can be considered to see the findings are still consistent. For instance, initially meet two metabolic syndrome components as MHO, now meet four.

**R2)**

Thank you for reviewer’s reasonable comments.

Because of the lack of universally accepted criteria to identify MHO, its prevalence varies widely among studies [1]. Moreover, the effects of MHO on lung function could be dependent on the criteria used, as reviewer is concerned. So, the reviewer makes a very important point. We fully understand reviewer’s concern.

As reviewer pointed out, we did sensitivity analysis of more strict definition criteria of metabolic health that used in previous studies [2-4]. We defined MH (metabolically healthy) persons as those having none of metabolic abnormalities that we showed in current manuscript. We think that this more strict definition of MH may allow us to identify the effect of MH on lung function that was possibly missed in the current study.

The same associations between MHO and lung function was also identified in the analysis with use of more strict definition of MHO, as we previously reported. However, the aOR results were attenuated, compared to the original results. Although the reasons for these attenuation are unclear, change in proportion of HOMA-IR ≥ 90th percentile may be involved. As more strict definition criteria of MH was applied, HOMA-IR was reduced within four phenotypes classified by metabolic health and obesity status, especially in subjects with metabolically unhealthy phenotypes. This phenomenon could be caused by shift towards MHU group of subjects with low burden of metabolic abnormalities.

|  | MHNO | MHO | MUHNO | MUHO |
| --- | --- | --- | --- | --- |
| HOMA-IR  Original definition  More strict definition | 1.13 (0.8-1.55)  1.06 (0.75-1.44) | 1.54 (1.15-2.02)  1.37 (1.03-1.78) | 1.96 (1.35-2.70)  1.54 (1.09-2.19) | 2.73 (1.98-3.65)  2.33 (1.64-3.22) |
| HOMA-IR≥90^th^ percentile  Original definition  More strict definition | 635 (0.9%)  0 (0%) | 797 (4.6%)  0 (0%) | 2,256 (18.8%)  2,894 (9.3%) | 7,794 (44.1%)  8,591 (32.2%) |

Insulin resistance is an underlying factor for metabolic syndrome [5] and also the common pathways underlying lung function impairment and metabolic syndrome.[5-7] Insulin resistance reduces glucose utilization and induces abnormal fat metabolism in skeletal muscle, which may impair mitochondrial ATP production and reduce skeletal muscle strength [8]. As forced respiration during spirometry requires respiratory skeletal muscle contraction, insulin resistance could mediate a decline of lung function. Therefore, attenuated aOR results could be possible in the analysis with use of more strict definition of MHO, as we previously reported.

The specific result is following;

**Multiple logistic regression analysis of impaired spirometric parameters according to metabolic health and obesity status.**

|  | Model 1 | | | Model 2 | | | Model 3 | | |
| --- | --- | --- | --- | --- | --- | --- | --- | --- | --- |
|  | OR (95% CI) | p value | p for trend | OR (95% CI) | p value | p for trend | OR (95% CI) | p value | p for trend |
| FEV1%< 80% |  |  | <0.001 |  |  | <0.001 |  |  | 0.001 |
| MHNO (reference) | 1 |  |  | 1 |  |  | 1 |  |  |
| MHO | 0.816 (0.681-0.977) | 0.027 |  | 0.787 (0.653-0.948) | 0.012 |  | 0.785 (0.651-0.946) | 0.011 |  |
| MUHNO | 1.268(1.149-1.399) | <0.001 |  | 1.198 (1.081-1.328) | 0.001 |  | 1.191 (1.073-1.323) | 0.001 |  |
| MUHO | 1.257 (1.105-1.431) | 0.001 |  | 1.115 (0.972-1.279) | 0.118 |  | 1.106 (0.961-1.272) | 0.159 |  |
| FVC%<80% |  |  | <0.001 |  |  | <0.001 |  |  | <0.001 |
| MHNO (reference) | 1 |  |  | 1 |  |  | 1 |  |  |
| MHO | 0.579 (0.463-0.723) | <0.001 |  | 0.545 (0.431-0.687) | <0.001 |  | 0.542 (0.429-0.684) | <0.001 |  |
| MUHNO | 1.255 (1.090-1.444) | 0.002 |  | 1.151 (0.992-1.337) | 0.064 |  | 1.136 (0.975-1.322) | 0.102 |  |
| MUHO | 1.197 (1.076-1.331) | 0.001 |  | 1.147 (1.026-1.282) | 0.016 |  | 1.137 (1.015-1.273) | 0.026 |  |
| FEV1(L)/FVC(L) ratio < 0.7 |  |  | 0.182 |  |  | 0.601 |  |  | 0.895 |
| MHNO (reference) | 1 |  |  | 1 |  |  | 1 |  |  |
| MHO | 1.026 (0.851-1.237) | 0.789 |  | 1.106 (0.908-1.347) | 0.315 |  | 1.144 (0.935-1.399) | 0.191 |  |
| MUHNO | 0.905 (0.710-1.153) | 0.419 |  | 0.938 (0.730-1.205) | 0.615 |  | 0.958 (0.750-1.233) | 0.741 |  |
| MUHO | 1.244 (1.048-1.478) | <0.013 |  | 1.285 (1.074-1.537) | 0.006 |  | 1.298 (1.085-1.554) | 0.004 |  |

Model 1 was adjusted for age, sex, smoking status, alcohol intake, regular exercise and education level. Model 2 was adjusted as in model 1 plus systolic BP, glucose, total cholesterol triglycerides, high-density lipoprotein cholesterol, low density lipoprotein cholesterol, and HOMA-IR. Model 3 was adjusted as in model 2 plus variables with a *p* value<0.05 in the univariate analyses. CI=confidence interval; FEV1%=percent predicted forced expiratory volume in 1s; FVC%=percent predicted forced vital capacity; MHNO=metabolically healthy non-obese; MHO=metabolically healthy obese; MUHNO=metabolically unhealthy non-obese; MUHO=metabolically unhealthy obese; OR=odds ratio.

**C3)**

The reporting of the study design can be better organized. Covariates, exposures, and outcomes should be described under the corresponding headings. For example, blood samples should be described under metabolic status, and covariates should be written with subheadings.

**R3)**

Thank you for the reviewer’s reasonable comments. The reviewer makes a very important point. We fully understand reviewer’s concern. We reflected reviewer’s recommendations, as seen from the modified manuscript file attached to this point by-point response.

We modified method section as follows, considering reviewer’s comment.

**(from line 74 to line 91 on page 5) in the revised manuscript.**

**Study design and population**

This cross-sectional study was a part of the Kangbuk Samsung Health Study (KSHS), in which subjects participated in a medical health checkup program at the Health Promotion Center of Kangbuk Samsung Hospital. In South Korea, the Industrial Safety and Health Law requires all employees to participate in annual or biennial health examinations that are offered free of charge. Most of the examinees were employees and family members of companies or local governmental organizations. The remaining participants voluntarily registered for screening examinations.

We initially included 214,551 individuals who underwent comprehensive health examinations in 2019. From these, we extracted 212,333 participants with recorded spirometry and metabolic trait data used to ascertain metabolic health. Among the potential subjects, we excluded subjects with missing data for medical history and smoking habits or alcohol consumption (n = 17,759). We additionally excluded 80,431 participants with either self-reported histories and/or who were currently receiving medications for any medical conditions. Detailed comorbidities were unavailable, because the medical history questionnaire only required yes/no responses. As some individuals had more than one exclusion criterion, 114,143 subjects were eligible for final analysis (Fig. 1).

🡪

**Study design and population**

This cross-sectional study was a part of the Kangbuk Samsung Health Study (KSHS), in which subjects participated in a medical health checkup program at the Health Promotion Center of Kangbuk Samsung Hospital. The comprehensive health-screening program assessed demographic, anthropometric, and laboratory data. In South Korea, the Industrial Safety and Health Law requires all employees to participate in annual or biennial health examinations that are offered free of charge. Most of the examinees were employees and family members of companies or local governmental organizations. The remaining participants voluntarily registered for screening examinations.

We initially included 214,551 individuals who underwent comprehensive health examinations in 2019. From these, we extracted 212,333 participants with recorded spirometry and metabolic trait data used to ascertain metabolic health. Among the potential subjects, we excluded subjects with missing data for medical history and smoking habits or alcohol consumption (n = 17,759). We additionally excluded 80,431 participants with either self-reported histories and/or who were currently receiving medications for any medical conditions. Detailed comorbidities were unavailable, because the medical history questionnaire only required yes/no responses. As some individuals had more than one exclusion criterion, 114,143 subjects were eligible for final analysis (Fig. 1).

**(from line 102 to line 121 on page 6-7 ) in the revised manuscript.**

**Data collection, anthropometric measurements, and laboratory tests**

Physical characteristics and serum biochemical parameters were measured by trained nurses. Height and weight were calculated with individuals wearing light clothing and bare feet , using automated instruments (InBody 3.0 and Inbody 720, Biospace Co., Seoul, Korea) that were validated for reproducibility and accuracy of body composition measurements [9] and calibrated every morning before testing started. Body mass index (BMI) was calculated as weight in kg divided by height in m^2^. Blood pressure (BP) was measured with a standard sphygmomanometer after at least 5 min of seated rest. Measurements were performed twice at 5 min intervals and averaged for analysis.

Blood samples were taken after a 10 h fast. Methods for measuring serum levels of glucose, lipid profiles, liver enzymes, creatinine, and high-sensitivity C-reactive protein (hsCRP) have been described previously [10,11]. The inter- and intraassay coefficients of variation for quality control specimens were <5% for the blood variables. Insulin resistance was assessed using the homeostasis model assessment of insulin resistance (HOMA-IR) equation: fasting blood insulin (μU/ml) × fasting blood glucose (mmol/l)/22.5 [12]. The Laboratory Medicine Department at Kangbuk Samsung Hospital has been accredited and participates annually in inspections and surveys by the Korean Association of Quality Assurance for Clinical Laboratories.

🡪

**Exposures: Metabolic health and obesity status**

Physical characteristics and serum biochemical parameters were measured by trained nurses. Height and weight were calculated with individuals wearing light clothing and bare feet , using automated instruments (InBody 3.0 and Inbody 720, Biospace Co., Seoul, Korea) that were validated for reproducibility and accuracy of body composition measurements [9] and calibrated every morning before testing started. Body mass index (BMI) was calculated as weight in kg divided by height in m^2^. Blood pressure (BP) was measured with a standard sphygmomanometer after at least 5 min of seated rest. Measurements were performed twice at 5 min intervals and averaged for analysis.

Blood samples were taken after a 10 h fast. Serum total cholesterol and triglycerides were determined with an enzymatic colorimetric assay. Low-density lipoprotein cholesterol (LDL-C) and HDL-C were measured directly with a homogeneous enzymatic colorimetric assay. Serum glucose was measured by the hexokinase method on a Cobas Integra 800 apparatus (Roche Diagnostics). The inter- and intraassay coefficients of variation for quality control specimens were <5% for the blood variables. Insulin resistance was assessed using the homeostasis model assessment of insulin resistance (HOMA-IR) equation: fasting blood insulin (μU/ml) × fasting blood glucose (mmol/l)/22.5 [12] The Laboratory Medicine Department at Kangbuk Samsung Hospital has been accredited and participates annually in inspections and surveys by the Korean Association of Quality Assurance for Clinical Laboratories.

And, we moved the paragraph with subheadings “Definition of metabolic health and obesity status “ into the subheadings, Exposures: Metabolic health and obesity status

**(from line 121 to line 130 on page 7) in the revised manuscript.**

**Definition of metabolic health and obesity status**

Obesity was defined as BMI ≥25 kg/m^2^ (the proposed cut-off for Asian populations) [4,10,11,13]. Metabolic health was defined as having less than two of the following risk factors [14]; elevated blood pressure ≥ 130/85) or anti-hypertensive drugs, high triglyceride (≥150 mg/dl) or lipid-lowering drugs, high fasting glucose (≥100 mg/dl) or medications for diabetes, low HDL-cholesterol (<40 mg/dL in men, <50 mg/dL in women), and HOMA-IR ≥90th percentile (≥2.9). We used this definition alongside obesity status to create four phenotypes: metabolically healthy, non-obese (MHNO), metabolically healthy, obese (MHO), metabolically unhealthy, non-obese (MUHNO) and metabolically unhealthy, obese (MUHO).

🡪

Insulin resistance was assessed using the homeostasis model assessment of insulin resistance (HOMA-IR) equation: fasting blood insulin (μU/ml) × fasting blood glucose (mmol/l)/22.5 [12] The Laboratory Medicine Department at Kangbuk Samsung Hospital has been accredited and participates annually in inspections and surveys by the Korean Association of Quality Assurance for Clinical Laboratories. Obesity was defined as BMI ≥25 kg/m^2^ (the proposed cut-off for Asian populations) [4,10,11,13]. Metabolic health was defined as having less than two of the following risk factors [14]; elevated blood pressure ≥ 130/85) or anti-hypertensive drugs, high triglyceride (≥150 mg/dl) or lipid-lowering drugs, high fasting glucose (≥100 mg/dl) or medications for diabetes, low HDL-cholesterol (<40 mg/dL in men, <50 mg/dL in women), and HOMA-IR ≥90th percentile (≥2.9). Metabolically unhealthy (MUH) was defined as having two or more of the metabolic abnormalities described above. We used this definition alongside obesity status to create four phenotypes: metabolically healthy, non-obese (MHNO), metabolically healthy, obese (MHO), metabolically unhealthy, non-obese (MUHNO) and metabolically unhealthy, obese (MUHO).

**(from line 132 to line 143 on page 7) in the revised manuscript.**

**Data collection, anthropometric measurements, and laboratory tests**

The comprehensive health-screening program assessed demographic, anthropometric, and laboratory data. Information on demographic characteristics, smoking habits, alcohol intake (g/day), exercise frequency, medical history, medication use and education level were acquired using standardized, self-administered questionnaires. Smoking status was classified as nonsmoker, ex-smokers (any prior regular use), and current smokers (current use). Alcohol consumption was categorized as non-heavy (≤20g/day) and heavy (>20g/day). Weekly frequency of moderate physical activity (defined as more than 30 min of activity per day inducing slight breathlessness) was also assessed, and regular exercise was defined as ≥3 times/week [4]. Education level was categorized as less than college graduate or college graduate or more [4].

🡪

**Covariates**

Our analyses were adjusted for socio-demographic variables, behavioral factors and laboratory parameters. Information on demographic characteristics, smoking habits, alcohol intake (g/day), exercise frequency, medical history, medication use and education level were acquired using standardized, self-administered questionnaires. Smoking status was classified as nonsmoker, ex-smokers (any prior regular use), and current smokers (current use). Alcohol consumption was categorized as non-heavy (≤20g/day) and heavy (>20g/day). Weekly frequency of moderate physical activity (defined as more than 30 min of activity per day inducing slight breathlessness) was also assessed, and regular exercise was defined as ≥3 times/week [4]. Education level was categorized as less than college graduate or college graduate or more [4]. Additionally, serum levels of liver enzymes, creatinine, and high-sensitivity C-reactive protein (hsCRP) were measured as described previously [10,11].

**(from line 145 to line 156 on page 8) in the revised manuscript.**

**Lung function measurement**

Spirometry was performed as recommended by the American Thoracic Society [15] using the Vmax22 system (Sensor-Medics, Yorba Linda, CA). FEV1 and FVC were obtained under a pre-bronchodilatory setting. The highest forced expiratory volume in 1s (FEV1) and forced vital capacity (FVC) values from three or more tests with acceptable curves were used for further analyses. The predicted values for FEV1 and FVC were calculated using equations for a representative Korean population sample [16]. To calculate the predicted FVC% (FVC%) and predicted FEV1% (FEV1%), we divided the measured value (L) by the predicted value (L) and converted the quotient into a percentage. The ratio of FEV1 to FVC (FEV1/FVC) was also calculated, where the actual measurement was used. The following criteria were used to determine impaired lung function: FEV1% < 80%, FVC% < 80%, and FEV1/FVC < 0.7 (refer to obstructive lung function, OLF) [17].

**🡪**

**Outcomes: Lung function**

Spirometry was performed as recommended by the American Thoracic Society [15] using the Vmax22 system (Sensor-Medics, Yorba Linda, CA). FEV1 and FVC were obtained under a pre-bronchodilatory setting. The highest forced expiratory volume in 1s (FEV1) and forced vital capacity (FVC) values from three or more tests with acceptable curves were used for further analyses. The predicted values for FEV1 and FVC were calculated using equations for a representative Korean population sample [16]. To calculate the predicted FVC% (FVC%) and predicted FEV1% (FEV1%), we divided the measured value (L) by the predicted value (L) and converted the quotient into a percentage. The ratio of FEV1 to FVC (FEV1/FVC) was also calculated, where the actual measurement was used. The following criteria were used to determine impaired lung function: FEV1% < 80%, FVC% < 80%, and FEV1/FVC < 0.7 (refer to obstructive lung function, OLF) [17].

**C4)**

Figure 2 can be presented in a table instead of a graph.

**R4)**

Thank you for reviewer’s advice. As reviewer suggested, we presented adjusted mean values of lung function parameters in a new table.

**Figure 2.** **Adjusted mean values of lung function parameters in the study group classified by metabolic health and obesity status.**

A) FEV1 (liter); B) FVC (liter); C) FEV1%; D) FVC%;E)FEV1 (liter)/FVC (liter) ratio. Data are presented as mean ± standard deviation. The multivariable model was adjusted for age, sex, and continuous variables with a p value*<*0.05 in the univariate analyses, Including systolic blood pressure, smoking (pack-years), amount of alcohol consumption (g/day) , moderate physical activity frequency (times/week), glucose, lipid profiles, liver enzymes, creatinine, high-sensitivity C-reactive and HOMA-IR.

FEV1% = percent predicted forced expiratory volume in 1s; FVC% = percent predicted forced vital capacity; HOMA-IR = homeostasis model assessment of insulin resistance; MHNO = metabolically healthy, non-obese; MHO = metabolically healthy, obese; MUHNO = metabolically unhealthy, non-obese; MUHO = metabolically unhealthy, obese.

🡪

During the revision process, we realized mistakes in figure 2-B. We apologize for our carelessness and the lack of clarity. The p value between MUHNO and MUHO should be “<0.001”, instead of “0.0.34” and also, the p value between MHNO and MUHO should be changed into “0.034” from “<0.001”. Furthermore, we originally described mean ± standard deviation for spirometric values. But data should be presented as adjusted mean ± standard error of mean.

**Table 2. Adjusted mean values of lung function parameters in the study group classified by metabolic health and obesity status.**

|  | **Category** | | | | ***p* value by ANCOVA** | **Adjusted *p* value^a^** | | | | | |
| --- | --- | --- | --- | --- | --- | --- | --- | --- | --- | --- | --- |
|  | **MHNO**  **(N = 67,261)**  **(58.9%)** | **MHO**  **(N = 17,192)**  **(15.1%)** | **MUHNO**  **(N = 12,005)**  **(10.5%)** | **MUHO**  **(N = 17,685)**  **(15.5%)** |  | **MHNO vs**  **MHO** | **MHNO vs**  **MUHNO** | **MHNO vs**  **MUHO** | **MHO vs**  **MUHNO** | **MHO vs**  **MUHO** | **MUHNO vs**  **MUHO** |
| **FEV1 (liter)** | 3.306±0.005 | 3.332±0.004 | 3.217±0.005 | 3.289±0.005 | <0.001 | <0.001 | <0.001 | 0.011 | <0.001 | <0.001 | <0.001 |
| **FEV1%** | 97.822±0.140 | 98.353±0.106 | 95.779±0.150 | 97.281±0.144 | <0.001 | 0.001 | <0.001 | 0.005 | <0.001 | <0.001 | <0.001 |
| **FVC (liter)** | 4.049±0.006 | 4.074±0.004 | 3.891±0.006 | 4.031±0.006 | <0.001 | <0.001 | <0.001 | 0.034 | <0.001 | <0.001 | <0.001 |
| **FVC%** | 98.883±0.142 | 99.311±0.104 | 95.593±0.148 | 98.354±0.138 | <0.001 | 0.004 | <0.001 | 0.005 | <0.001 | <0.001 | <0.001 |
| **FEV1(L)/FVC(L) ratio**  **FEV1(L)/FVC(L)x100** | 0.824±0.001  82.403±0.080 | 0.815±0.001  81.500±0.082 | 0.831±0.001  83.092±0.085 | 0.820±0.001  82.019±0.066 | <0.001  <0.001 | <0.001  <0.001 | <0.001  <0.001 | <0.001  <0.001 | <0.001  <0.001 | <0.001  <0.001 | <0.001  <0.001 |

Data are presented as adjusted mean ± standard error. The multivariable model was adjusted for age, sex, and continuous variables with a p value*<*0.05 in the univariate analyses, Including systolic blood pressure, smoking (pack-years), amount of alcohol consumption (g/day) , moderate physical activity frequency (times/week), glucose, lipid profiles, liver enzymes, creatinine, high-sensitivity C-reactive and HOMA-IR.

^a^Adjusted p value using a Bonferroni correction

FEV1% = percent predicted forced expiratory volume in 1s; FVC% = percent predicted forced vital capacity; HOMA-IR = homeostasis model assessment of insulin resistance; MHNO = metabolically healthy, non-obese; MHO = metabolically healthy, obese; MUHNO = metabolically unhealthy, non-obese; MUHO = metabolically unhealthy, obese.

And also we add a sentence to explain multiple comparison in statistical analysis as follows;

**(from line 167 to line 168 on page 8) in the revised manuscript.**

Analysis of covariance (ANCOVA) was performed to test differences of mean values of lung function parameters between study groups after adjusting for age, sex, and continuous variables with a p value*<*0.05 in the univariate analyses. To analyze the significance of differences among groups according to metabolic health and obesity status, all covariates were treated as categorical variables: high or low and with or without. Differences among groups were tested using chi square tests or Fisher’s exact test.

🡪

Analysis of covariance (ANCOVA) was performed to test differences of mean values of lung function parameters between study groups after adjusting for age, sex, and continuous variables with a p value*<*0.05 in the univariate analyses. Post-hoc analysis was performed using the Bonferroni correction, comparing the mean spirometric values between study groups. To analyze the significance of differences among groups according to metabolic health and obesity status, all covariates were treated as categorical variables: high or low and with or without. Differences among groups were tested using chi square tests or Fisher’s exact test.

**C5)**

The definition of metabolic derangement should be provided.

**R5)**

Thank you for pointing out the lack of clarity. However, we think that it would be better to replace “metabolic derangement” with “metabolically unhealthy” for consistency of context.

And also we changed “metabolic derangement” into “metabolically unhealthy” in the manuscript and tables. As reviewer pointed out, we clarified definition of “metabolically unhealthy” at method section as follows;

**(from line 126 to line 127 on page 7) in the revised manuscript.**

Metabolic health was defined as having less than two of the following risk factors [14]; elevated blood pressure ≥ 130/85) or anti-hypertensive drugs, high triglyceride (≥150 mg/dl) or lipid-lowering drugs, high fasting glucose (≥100 mg/dl) or medications for diabetes, low HDL-cholesterol (<40 mg/dL in men, <50 mg/dL in women), and HOMA-IR ≥90th percentile (≥2.9). We used this definition alongside obesity status to create four phenotypes: metabolically healthy, non-obese (MHNO), metabolically healthy, obese (MHO), metabolically unhealthy, non-obese (MUHNO) and metabolically unhealthy, obese (MUHO).

🡪

Metabolic health was defined as having less than two of the following risk factors [14]; elevated blood pressure ≥ 130/85) or anti-hypertensive drugs, high triglyceride (≥150 mg/dl) or lipid-lowering drugs, high fasting glucose (≥100 mg/dl) or medications for diabetes, low HDL-cholesterol (<40 mg/dL in men, <50 mg/dL in women), and HOMA-IR ≥90th percentile (≥2.9). Metabolically unhealthy (MUH) was defined as having two or more of the metabolic abnormalities described above. We used this definition alongside obesity status to create four phenotypes: metabolically healthy, non-obese (MHNO), metabolically healthy, obese (MHO), metabolically unhealthy, non-obese (MUHNO) and metabolically unhealthy, obese (MUHO).

**References**

**1.**	Velho S, Paccaud F, Waeber G, Vollenweider P, Marques-Vidal P (2010) Metabolically healthy obesity: different prevalences using different criteria. Eur J Clin Nutr 64: 1043-1051.

**2.**	Chang Y, Ryu S, Choi Y, Zhang Y, Cho J, Kwon MJ, et al. (2016) Metabolically Healthy Obesity and Development of Chronic Kidney Disease: A Cohort Study. Ann Intern Med 164: 305-312.

**3.**	Kim TJ, Shin HY, Chang Y, Kang M, Jee J, Choi YH, et al. (2017) Metabolically healthy obesity and the risk for subclinical atherosclerosis. Atherosclerosis 262: 191-197.

**4.**	Chang Y, Kim BK, Yun KE, Cho J, Zhang Y, Rampal S, et al. (2014) Metabolically-healthy obesity and coronary artery calcification. J Am Coll Cardiol 63: 2679-2686.

**5.**	Gallagher EJ, Leroith D, Karnieli E (2010) Insulin resistance in obesity as the underlying cause for the metabolic syndrome. Mt Sinai J Med 77: 511-523.

**6.**	Lim SY, Rhee EJ, Sung KC (2010) Metabolic syndrome, insulin resistance and systemic inflammation as risk factors for reduced lung function in Korean nonsmoking males. J Korean Med Sci 25: 1480-1486.

**7.**	Lin WY, Yao CA, Wang HC, Huang KC (2006) Impaired lung function is associated with obesity and metabolic syndrome in adults. Obesity (Silver Spring) 14: 1654-1661.

**8.**	Barzilay JI, Cotsonis GA, Walston J, Schwartz AV, Satterfield S, Miljkovic I, et al. (2009) Insulin resistance is associated with decreased quadriceps muscle strength in nondiabetic adults aged >or=70 years. Diabetes Care 32: 736-738.

**9.**	Malavolti M, Mussi C, Poli M, Fantuzzi AL, Salvioli G, Battistini N, et al. (2003) Cross-calibration of eight-polar bioelectrical impedance analysis versus dual-energy X-ray absorptiometry for the assessment of total and appendicular body composition in healthy subjects aged 21-82 years. Ann Hum Biol 30: 380-391.

**10.**	Rhee EJ, Lee MK, Kim JD, Jeon WS, Bae JC, Park SE, et al. (2014) Metabolic health is a more important determinant for diabetes development than simple obesity: a 4-year retrospective longitudinal study. PLoS One 9: e98369.

**11.**	Rhee EJ, Seo MH, Kim JD, Jeon WS, Park SE, Park CY, et al. (2013) Metabolic health is more closely associated with coronary artery calcification than obesity. PLoS One 8: e74564.

**12.**	Matthews DR, Hosker JP, Rudenski AS, Naylor BA, Treacher DF, Turner RC (1985) Homeostasis model assessment: insulin resistance and beta-cell function from fasting plasma glucose and insulin concentrations in man. Diabetologia 28: 412-419.

**13.**	Jung CH, Lee WJ, Song KH (2017) Metabolically healthy obesity: a friend or foe? Korean J Intern Med 32: 611-621.

**14.**	Wildman RP, Muntner P, Reynolds K, McGinn AP, Rajpathak S, Wylie-Rosett J, et al. (2008) The obese without cardiometabolic risk factor clustering and the normal weight with cardiometabolic risk factor clustering: prevalence and correlates of 2 phenotypes among the US population (NHANES 1999-2004). Arch Intern Med 168: 1617-1624.

**15.**	(1995) Standardization of Spirometry, 1994 Update. American Thoracic Society. Am J Respir Crit Care Med 152: 1107-1136.

**16.**	Choi HS, Park YB, Yoon HK, Lim SY, Kim TH, Park JH, et al. (2019) Validation of Previous Spirometric Reference Equations and New Equations. J Korean Med Sci 34: e304.

**17.**	Miller MR, Hankinson J, Brusasco V, Burgos F, Casaburi R, Coates A, et al. (2005) Standardisation of spirometry. Eur Respir J 26: 319-338.
