# Supplementary material for: The effect of metabolic health and obesity on lung function: A cross sectional study of 114,143 participants from Kangbuk Samsung Health Study
Source: PLoS One. 2022 Apr 13;17(4):e0266885. doi: 10.1371/journal.pone.0266885 (PMC9007386; doi:10.1371/journal.pone.0266885)
Supplement: Attachment — Submitted filename: Response to Reviewers.docx [file pone.0266885.s003.docx]

Manuscript ID: PONE-D-21-23871

**Title: The effect of metabolic health and obesity on lung function:**

**A cross sectional study of 114,143 participants from Kangbuk Samsung Health Study**

The authors appreciate the time and effort of the reviewers and the editor in reviewing this manuscript and in providing thoughtful and helpful comments. We are pleased to have an opportunity to improve our paper. The constructive criticism has been well-received by all of us. In response to the reviewer’s suggestions and questions, we have revised the manuscript as suggested. We have responded to the reviewer’s comments in a point-by-point manner below. We have included the changes recommended by the editors and reviewers in the revised manuscript (in red). Please find enclosed the revised version of the manuscript. We believe that we have dealt with all reviewer comments and hope that this work is now suitable for publication.

We look forward to your response.

With best wishes

Jae-Uk Song, MD, PhD,

Division of Pulmonary and Critical Care Medicine, Department of Internal Medicine, Kangbuk Samsung Hospital, Sungkyunkwan University School of Medicine,

29 Saemunan-ro, Jongno-gu, Seoul 03181, Republic of Korea

Phone: +82-2-2001-1597

Fax: +82-2-2001-1596

E-mail: [khfever76@gmail.com](mailto:khfever76@gmail.com)

**Point-by-point responses to reviewer’s comments**

**TO THE COMMENTS OF JOURNAL REQUIREMENTS**

**C1)**

Please review your reference list to ensure that it is complete and correct. If you have cited papers that have been retracted, please include the rationale for doing so in the manuscript text, or remove these references and replace them with relevant current references. Any changes to the reference list should be mentioned in the rebuttal letter that accompanies your revised manuscript. If you need to cite a retracted article, indicate the article’s retracted status in the References list and also include a citation and full reference for the retraction notice.

**R1)**

During the second revision, we did not retract, remove, or replace cited papers.

There was no change to the reference list. We reviewed our references to ensure that they are complete and correct. Thank you for your concerns.

**TO THE COMMENTS OF THE REVIEWER 1.**

Thanks for the authors’ revision. The author has addressed all the points I mentioned before and interpreted them reasonably and comprehensively. There are some minor comments attached

**Minor comments**

**C1)**

 It would be better to do some English proofreading to make the manuscript easier to read.

**R1)**

We appreciate the reviewer’s advice and suggestions. The manuscript was revised by a commercial English-language editing service (eWorld Editing, Eugene, OR, USA). Changes were made after correction of English by a native English speaker, and these parts is marked in red. Please find enclosed the revised version of the manuscript. We also attached the certification of proofreading from eWorld Editing, Eugene, OR, USA.

**C2)**

As for R2, the paragraph simply explains why a stricter definition would give the most conservative result. It would be transparent to show the readers if the OR would still be relevant or even stronger

**R2)**

Thank you for the comments. This is a very important point.

The marked heterogeneity of MHO definitions could contribute in part to the inconsistencies found in the association of MHO with lung function. Thus, similar results from analysis using another definition of MH is important to support our results. Therefore, we added the following table in the supporting information. We added a paragraph in the Discussion section explaining why a stricter definition would produce stronger aOR values of MHO for impaired lung function and attenuated aOR values of MUHNO and MUHO compared to those from the analysis using a less strict definition. We hope this addresses your concerns.

**(from line 286 to line 301 on page 24) in the revised manuscript**

Furthermore, the same association between MHO and lung function was identified using a strict definition of MH as absence of any component of metabolic syndrome [1]. The aOR was attenuated in MUHNO and MUHO in contrast to a stronger aOR value of MHO compared to the original results (S1 Table). The reasons for these results are unclear. However, individuals with MH phenotypes could be more homogeneous, and vice versa when using a strict definition of MH. These changes seemed to impact the effect of MH on lung function more strictly despite an attenuated effect of MUH on lung function. Consequently, a strict definition of MH might have allowed us to identify effects of metabolic health status on lung function that were missed when using the definition of MH as having fewer than 2 metabolic components, because each metabolic parameters is a risk factor of decreased lung function. [2,3] Therefore, the aOR values might be stronger in the MHO phenotype and weaker in MUHNO and MUHO. These changes would provide more relevant results, as in a previous study assessing the effect of MHO on coronary calcium. [1] Such findings are important to support the benefit of MHO for lung function, because the marked heterogeneity of MHO definitions could cause differences in the association with lung function.

We also modified some sentences in the discussion section as follows.

**(from line 259 to line 268 on page 23) in the revised manuscript**

The prevalence of MHO greatly varies (10-51%), according to race and the criteria used to define MHO [4]. MHO is more common in women, younger individuals, and Asian populations compared with Europeans or individuals of multiethnic origin [4]. The prevalence of MHO (15.1%) assessed using criteria proposed by Wildman et al. [5] was generally comparable to that (11.3-18.2%) reported in the Korean general population using the same criteria [6,7], whereas this proportion was lower than in a previous study (25.8%) using a less strict definition [8]. Additionally, other studies found lower estimates (7.9%) using a very strict definition [1]. Even in samples of the same ethnicity, MHO prevalence varies depending on the definition used. Thus, findings from studies of the association between MHO and clinical outcomes should be interpreted with caution. However, there is no consensus on the definition of MHO. The urgent establishment of a common definition of MHO is necessary to clarify the impact of MHO on clinical outcomes.

🡪

The prevalence of MHO greatly varies (10-51%), according to race and the definition [4]. MHO is more common in women, younger individuals, and Asian populations compared with Europeans or individuals of multiethnic origin [4]. The prevalence of MHO (15.1%) assessed using criteria proposed by Wildman et al. [5] was comparable to that (11.3-18.2%) reported in the Korean general population using the same criteria [6,7], whereas this proportion was lower than in a previous study (25.8%) using a less strict definition [8]. Additionally, other studies found lower estimates (7.9%) using a very strict definition [1]. Even in samples of the same ethnicity, MHO prevalence varies depending on the definition. However, there is no consensus on the definition of MHO. Urgent establishment of a common definition of MHO is necessary to evaluate robustly its impact on clinical outcomes.

**(from line 373 to line 374 on page 27) in the revised manuscript**

Second, our results were obtained in a middle aged Korean health screening cohort with moderate obesity and use of MHO criteria proposed by Wildman et al. [5], which means it is not possible to generalize our findings. In particular, since there is no unified definition of metabolic health, there might be major differences in outcomes according to the definition applied.

🡪

Second, our results were obtained in a middle aged Korean health screening cohort with moderate obesity, which means it is not possible to generalize our findings.

**Multiple logistic regression analysis of impaired spirometric parameters according to metabolic health^a^ and obesity status.**

|  | Model 1 | | | Model 2 | | | Model 3 | | |
| --- | --- | --- | --- | --- | --- | --- | --- | --- | --- |
|  | OR (95% CI) | p value | p for trend | OR (95% CI) | p value | p for trend | OR (95% CI) | p value | p for trend |
| FEV1%< 80% |  |  | <0.001 |  |  | <0.001 |  |  | 0.001 |
| MHNO (reference) | 1 |  |  | 1 |  |  | 1 |  |  |
| MHO | 0.816 (0.681-0.977) | 0.027 |  | 0.787 (0.653-0.948) | 0.012 |  | 0.785 (0.651-0.946) | 0.011 |  |
| MUHNO | 1.268(1.149-1.399) | <0.001 |  | 1.198 (1.081-1.328) | 0.001 |  | 1.191 (1.073-1.323) | 0.001 |  |
| MUHO | 1.257 (1.105-1.431) | 0.001 |  | 1.115 (0.972-1.279) | 0.118 |  | 1.106 (0.961-1.272) | 0.159 |  |
| Metabolic unhealthy | 1.323 (1.215-1.44) |  | <0.001 | 1.236 (1.130-1.352) |  | <0.001 | 1.231 (1.123-1.350) |  | <0.001 |
| Obesity | 0.847 (0.793-0.903) |  | <0.001 | 0.904 (0.828-0.986) |  | 0.022 | 0.902 (0.820-0.993) |  | 0.035 |
| FVC%<80% |  |  | <0.001 |  |  | <0.001 |  |  | <0.001 |
| MHNO (reference) | 1 |  |  | 1 |  |  | 1 |  |  |
| MHO | 0.579 (0.463-0.723) | <0.001 |  | 0.545 (0.431-0.687) | <0.001 |  | 0.542 (0.429-0.684) | <0.001 |  |
| MUHNO | 1.255 (1.090-1.444) | 0.002 |  | 1.151 (0.992-1.337) | 0.064 |  | 1.136 (0.975-1.322) | 0.102 |  |
| MUHO | 1.197 (1.076-1.331) | 0.001 |  | 1.147 (1.026-1.282) | 0.016 |  | 1.137 (1.015-1.273) | 0.026 |  |
| Metabolic unhealthy | 1.347 (1.228-1.477) |  | <0.001 | 1.289 (1.168-1.421) |  | <0.001 | 1.279 (1.158-1.414) |  | <0.001 |
| Obesity | 0.863 (0.804-0.926) |  | <0.001 | 0.862 (0.785-0.947) |  | 0.002 | 0.835 (0.752-0.928) |  | 0.001 |
| FEV1(L)/FVC(L) ratio < 0.7 |  |  | 0.182 |  |  | 0.601 |  |  | 0.895 |
| MHNO (reference) | 1 |  |  | 1 |  |  | 1 |  |  |
| MHO | 1.026 (0.851-1.237) | 0.789 |  | 1.106 (0.908-1.347) | 0.315 |  | 1.144 (0.935-1.399) | 0.191 |  |
| MUHNO | 0.905 (0.710-1.153) | 0.419 |  | 0.938 (0.730-1.205) | 0.615 |  | 0.958 (0.750-1.233) | 0.741 |  |
| MUHO | 1.244 (1.048-1.478) | <0.013 |  | 1.285 (1.074-1.537) | 0.006 |  | 1.298 (1.085-1.554) | 0.004 |  |
| Metabolic unhealthy | 1.127 (0.993-1.279) |  | 0.065 | 1.116 (0.981-1.271) |  | 0.096 | 1.094 (0.958-1.250) |  | 0.183 |
| Obesity | 1.371 (1.212-1.550) |  | <0.001 | 1.139 (1.181-1.517) |  | <0.001 | 1.295 (1.131-1.484) |  | <0.001 |

**^a^**Metabolic health was defined as absence of any component of metabolic syndrome.

Model 1 was adjusted for age, sex, smoking status, alcohol intake, regular exercise and education level. Model 2 was adjusted as in model 1 plus systolic BP, glucose, total cholesterol, triglycerides, high-density lipoprotein cholesterol, low density lipoprotein cholesterol, and HOMA-IR. Model 3 was adjusted as in model 2 plus variables with a *p* <0.05 in univariate analyses. CI=confidence interval; FEV1%=percent predicted forced expiratory volume in 1s; FVC%=percent predicted forced vital capacity; MHNO=metabolically healthy non-obese; MHO=metabolically healthy obese; MUHNO=metabolically unhealthy non-obese; MUHO=metabolically unhealthy obese; OR=odds ratio.

**References**

**1.**	Chang Y, Kim BK, Yun KE, Cho J, Zhang Y, Rampal S, et al. (2014) Metabolically-healthy obesity and coronary artery calcification. J Am Coll Cardiol 63: 2679-2686.

**2.**	Leone N, Courbon D, Thomas F, Bean K, Jego B, Leynaert B, et al. (2009) Lung function impairment and metabolic syndrome: the critical role of abdominal obesity. Am J Respir Crit Care Med 179: 509-516.

**3.**	Lin WY, Yao CA, Wang HC, Huang KC (2006) Impaired lung function is associated with obesity and metabolic syndrome in adults. Obesity (Silver Spring) 14: 1654-1661.

**4.**	Jung CH, Lee WJ, Song KH (2017) Metabolically healthy obesity: a friend or foe? Korean J Intern Med 32: 611-621.

**5.**	Wildman RP, Muntner P, Reynolds K, McGinn AP, Rajpathak S, Wylie-Rosett J, et al. (2008) The obese without cardiometabolic risk factor clustering and the normal weight with cardiometabolic risk factor clustering: prevalence and correlates of 2 phenotypes among the US population (NHANES 1999-2004). Arch Intern Med 168: 1617-1624.

**6.**	Rhee EJ, Lee MK, Kim JD, Jeon WS, Bae JC, Park SE, et al. (2014) Metabolic health is a more important determinant for diabetes development than simple obesity: a 4-year retrospective longitudinal study. PLoS One 9: e98369.

**7.**	Rhee EJ, Seo MH, Kim JD, Jeon WS, Park SE, Park CY, et al. (2013) Metabolic health is more closely associated with coronary artery calcification than obesity. PLoS One 8: e74564.

**8.**	Choi KM, Cho HJ, Choi HY, Yang SJ, Yoo HJ, Seo JA, et al. (2013) Higher mortality in metabolically obese normal-weight people than in metabolically healthy obese subjects in elderly Koreans. Clin Endocrinol (Oxf) 79: 364-370.
